# Editorial: Grass Genome Evolution and Domestication

**DOI:** 10.3389/fpls.2022.866201

**Published:** 2022-04-05

**Authors:** Jorge Duitama, Laura E. Bartley, Romain Guyot, Rita Sharma

**Affiliations:** ^1^Department of Systems and Computing Engineering, Universidad de los Andes, Bogotá, Colombia; ^2^Institute of Biological Chemistry, Washington State University, Pullman, WA, United States; ^3^Institut de Recherche pour le Développement, UMR DIADE, Montpellier, France; ^4^Department of Biological Sciences, Birla Institute of Technology and Science (BITS), Pilani, India

**Keywords:** grasses, evolution, domestication, genomics, crops

Since their last common ancestor more than 100 million years ago, the grasses (*Poaceae*) have experienced a complex evolutionary history (Prasad et al., 2011). Whole genome duplications (WGDs), inter-species hybridization events, and large-scale rearrangements driven by mobile elements have all contributed to the enormous diversification of grass genomes (Qiao et al., 2019). At the end of this process, edible plant domestication through selection for important food production traits created new phenotypes and arguably new species. Grasses are one of the largest families within flowering plants, including the cereals which are crucial for food security.

The availability of long-read sequencing protocols and bioinformatic tools for genome assembly allows the reconstruction of entire genomes for a large number of species. In addition, the low cost of short-read sequencing allows researchers to acquire knowledge on large data sets, ranging from thousands of accessions of the same species to several dozen species of different genera. Together these data provide novel resources to study the evolution of genomes and genes across phylogenetic groups.

The articles collected in this Research Topic report new developments with relevance to genome evolution for *Poaceae* species in three major clades. Seetharam et al. present a chromosome-level assembly and annotation of the *Streptochaeta angustifolia* genome. Sister to all other major clades of grasses, the genome assembly of this species is an important tool that can serve as an outgroup within grasses for the research on evolution and diversification. The manuscript shows how the genome has a large collinearity with the rice genome, and that the *rho* WGD event was basal to grasses, including the Anomochlooideae subfamily, to which *S. angustifolia* belongs. Independent evolution, including several gene losses, appear to have produced the characteristic inflorescences in this species.

Two articles provide novel information on the evolution and domestication of *Oryza* species, to which the critical food crop, rice, belongs. Taking advantage of next-generation sequencing (NGS) data, He et al. describe the analysis of 1,464 plastid genomes from cultivated and wild rice accessions (*O. sativa* and *O. rufipogon*, respectively), thus providing an important resource for understanding the history and evolution of rice domestication, molecular dating, phylogeographic analyses and reconstruction of population historical dynamics. The analysis of the plastid genomes suggests a single domestication event for the *japonica* rice subspecies, and multiple events for the *indica* rice subspecies. In Pabuayon et al. parental and recombinant inbred lines of rice were analyzed through a multiomics approach to identify mechanisms of transgressive segregation for salinity tolerance. This study generates hypotheses for how synergy between phenotypes could lead to the genetic structure of cultivated species during the domestication process.

Using a low-depth short read sequencing strategy, Chalopin et al. present a comprehensive analysis of plastome and mobile element information for 51 bamboo species representing the three tribes of the Bambusoideae subfamily. Evidence of ancient hybridization and allopolyploidy explains previous disagreements between the analysis based on separate nuclear and plastid sequences. The authors propose biased fractionation and diploidization as important factors in the evolution of Bambusoideae species.

The role of polyploidy in the evolution and diversification of grass genomes is evident from the fact that most Poaceae are polyploid (Levy and Feldman, 2002). The allohexaploid genus *Kengyllia*, within the tribe Triticeae, to which wheat belongs, is a well characterized system for deciphering grass evolution through polyploidization. The *Kengyllia* species arose from hybridization of three ancestral diploid species with distinct genomes: St, P, and Y. The maternal lineages of *Kengyilia* species remained unresolved due to low sequence divergence in chloroplast and mitochondrial sequences. Chen et al. report a high-resolution phylogenetic analysis from 56 sequenced plastomes comprising 11 *Kengyilia* species and 45 related tetraploid and diploid Triticeae taxa. The genetic divergence pattern suggests both *Roegneria* and *Agropyron* tetraploids as maternal donors for *Kengylia*, supporting the independent origin of *Kengyilia* polyploid species.

Studying another genus within the tribe Triticeae, Xiong et al. report a complete *Elymus sibiricus* mitogenome assembly using long read sequencing data. The authors then use this in combination with other mitogenomes to reconstruct phylogenetic relationships and identify horizontal gene transfer events within flowering plant genomes.

Finally, two manuscripts describe new findings on the evolution of two important crop species within the Panicoideae subfamily, sorghum and sugarcane. First, Burgarella et al. present the sequencing and comparative analysis of the transcriptomes of 11 domesticated and 9 wild accessions of *Sorghum*. The study identified genes and pathways differentially expressed between wild and cultivated accessions. The patterns of expression suggest that the domestication process not only affects the variability of a few genes, but also produces an important rewiring of the transcriptome. Trujillo-Montenegro et al. present the genome assembly of a hybrid commercial variety of sugarcane. This species is particularly complex because it has an allodecaploid genome with *Saccarum spontaneous* and *Saccarum officinarum* as parental species. Ortholog identification, synteny and nucleotide evolution analyses across the gene space inform about genome evolution within the *Saccharum* genus and the divergence with *Sorghum*. Analysis of RNA-seq data revealed differentially expressed genes related to tolerance to drought and flood.

The manuscripts included in this Research Topic not only provide a significant amount of new information on the major clades of Poaceae, but also highlight the wide range of species that need to be covered to more fully understand the evolution of genomes within grasses. Genome-scale sequencing data for many taxa has begun to decipher the origin and reticulate evolution of grass genomes. Data from the huge and complex polyploid nuclear genomes, as well as from the transcriptomes, help to infer genetic divergence due to domestication and its impact on rewiring of genetic networks and consequent metabolic pathways, respectively. On the other hand, non-recombinant organellar genomes, due to their relative simplicity, uniparental inheritance, and evolutionary significance, continue to be primary targets for resolving lineage relationships across taxa. The studies reported in this Research Topic also demonstrate that integrating nuclear and organellar genomes can further enhance our ability to reconstruct evolutionary histories.

## Author Contributions

All authors listed have made a substantial, direct, and intellectual contribution to the work and approved it for publication.

## Conflict of Interest

The authors declare that the research was conducted in the absence of any commercial or financial relationships that could be construed as a potential conflict of interest.

## Publisher's Note

All claims expressed in this article are solely those of the authors and do not necessarily represent those of their affiliated organizations, or those of the publisher, the editors and the reviewers. Any product that may be evaluated in this article, or claim that may be made by its manufacturer, is not guaranteed or endorsed by the publisher.

## References

[B1] Levy A. A. Feldman M. (2002). The impact of polyploidy on grass genome evolution. Plant Physiol. 130, 1587–1593. 10.1104/pp.015727 12481041 PMC1540263

[B2] Prasad V. Strömberg C. Leaché A. Samant B. Patnaik R. Tang L.. (2011). Late cretaceous origin of the rice tribe provides evidence for early diversification in poaceae. Nat. Commun. 2, 480. 10.1038/ncomms1482 21934664

[B3] Qiao X. Li Q. Yin H. Qi K. Li L. Wang R.. (2019). Gene duplication and evolution in recurring polyploidization–diploidization cycles in plants. Genome Biol. 20, 38. 10.1186/s13059-019-1650-2 30791939 PMC6383267

